# Abnormal CFTR Affects Glucagon Production by Islet α Cells in Cystic Fibrosis and Polycystic Ovarian Syndrome

**DOI:** 10.3389/fphys.2017.00835

**Published:** 2017-11-17

**Authors:** Wen Qing Huang, Jing Hui Guo, Chun Yuan, Yu Gui Cui, Fei Yang Diao, Mei Kuen Yu, Jia Yin Liu, Ye Chun Ruan, Hsiao Chang Chan

**Affiliations:** ^1^Epithelial Cell Biology Research Centre, School of Biomedical Sciences, Faculty of Medicine, The Chinese University of Hong Kong, Hong Kong, Hong Kong; ^2^Department of Pathophysiology, Key Laboratory of State Administration of Traditional Chinese Medicine of the People's Republic of China, School of Medicine, Jinan University, Guangzhou, China; ^3^State Key Laboratory of Reproductive Medicine, Clinical Center of Reproductive Medicine, First Affiliated Hospital, Nanjing Medical University, Nanjing, China; ^4^Department of Biomedical Engineering, Faculty of Engineering, The Hong Kong Polytechnic University, Hong Kong, Hong Kong

**Keywords:** CFTR, glucagon, islet α cell, cystic fibrosis, PCOS

## Abstract

Glucagon, produced by islet α cells, functions to increase blood glucose. Abnormal glucose levels are often seen in cystic fibrosis (CF), a systematic disease caused by mutations of the CF transmembrane conductance regulator (CFTR), and in polycystic ovarian syndrome (PCOS), an endocrine disorder featured with hyperandrogenism affecting 5–10% women of reproductive age. Here, we explored the role of CFTR in glucagon production in α cells and its possible contribution to glucagon disturbance in CF and PCOS. We found elevated fasting glucagon levels in CFTR mutant (DF508) mice compared to the wildtypes. Glucagon and prohormone convertase 2 (PC2) were also upregulated in CFTR inhibitor-treated or DF508 islets, as compared to the controls or wildtypes, respectively. Dihydrotestosterone (DHT)-induced PCOS rats exhibited significantly lower fasting glucagon levels with higher CFTR expression in α cells compared to that of controls. Treatment of mouse islets or αTC1-9 cells with DHT enhanced CFTR expression and reduced the levels of glucagon and PC2. The inhibitory effect of DHT on glucagon production was blocked by CFTR inhibitors in mouse islets, and mimicked by overexpressing CFTR in αTC1-9 cells with reduced phosphorylation of the cAMP/Ca^2+^ response element binding protein (p-CREB), a key transcription factor for glucagon and PC2. These results revealed a previously undefined role of CFTR in suppressing glucagon production in α-cells, defects in which may contribute to glucose metabolic disorder seen in CF and PCOS.

## Introduction

Glucagon, a 29 amino acid peptide hormone produced by pancreatic α cells, functions to increase blood glucose, opposing the actions of insulin in the peripheral tissues (Jiang and Zhang, 2003). Its dysregulation is considered to underlie insulin deficiency, resulting in glucose metabolic disorders, such as diabetes, hypoglycemia (Cryer, 2002; Unger and Cherrington, 2012). Glucagon is cleaved by the prohormone convertase 2 (PC2) from proglucagon, which is encoded by the glucagon gene, in α cells (Rouillé et al., 1994; Piro et al., 2014). Proglucagon is also expressed in intestinal L cells or the neurons to produce glucagon-like peptide-1/2 (GLP-1/2) or oxyntomodulin by PC1/3 (Itoh et al., 1996; Nie et al., 2000; Wideman et al., 2007). Therefore, glucagon production relies on the gene transcription, as well as the enzyme conversion processes. The transcription of glucagon gene can be activated by a series of transcription factors, including the phosphorylated cAMP/Ca^2+^ response element binding protein (p-CREB) and paired box protein (Pax6) (Sander et al., 1997; Gosmain et al., 2010, 2011, 2012). The expression of PC2, the required enzyme for glucagon producing, also depends on its promoter binding with p-CREB (Espinosa et al., 2008). Thus, p-CREB is a master factor for glucagon production at gene transcription level.

Cystic fibrosis transmembrane conductance regulator (CFTR) is a cAMP-regulated anion channel conducting Cl^−^ (Sheppard and Welsh, 1999) and ${\text{HCO}}_{3}^{-}$ (Illek et al., 1997; Chen et al., 2012), which belongs to the superfamily of ATP binding cassette (ABC) transporter (Gadsby et al., 2006). CF-related diabetes (CFRD) is the most common comorbidity in subjects with CF (Moran et al., 2010), which caused by mutations of CFTR gene (Proesmans et al., 2008). Similarly, the polycystic ovarian syndrome (PCOS) patients also have high risk suffering from glucose metabolic disorders (Moran et al., 2011; Gambineri et al., 2012). PCOS is an endocrine disease affecting 5–10% of women in reproductive age (Norman et al., 2007; Goodarzi et al., 2011; Chen et al., 2012), featured with hyperandrogenism, insulin resistance, obesity and high risk of diabetes (Apridonidze et al., 2005; Fica et al., 2008; Galluzzo et al., 2008; Alpanés et al., 2014). Although glucose metabolism is known to be defective in both CFRD (Barrio, 2015; Koivula et al., 2016) and PCOS (Peppard et al., 2001; Moran et al., 2011), the exact underlying mechanism remains poorly understood.

We have recently discovered a novel role of CFTR in pancreatic islet β cells and insulin secretion, defect of which results in impaired and delayed glucose-induced insulin secretion, as observed in CFRD patients (Guo et al., 2014). It has also been reported that CFTR is localized in rat glucagon-secreting α cells (Boom et al., 2007; Edlund et al., 2017) and disrupted glucagon level is also observed in CFRD patients (Hinds et al., 1991; Lanng et al., 1993; Edlund et al., 2017), suggesting possible involvement of CFTR in the regulation of glucagon production; however, its exact role in pancreatic islet α cells remains unknown. Interestingly, CFTR expression can be upregulated by testosterone in prostate cancer (Xie et al., 2013). In PCOS, the fasting blood glucagon concentration is reported to be inversely related to androgen levels (Golland et al., 1990). Together with the findings that CFTR modulates p-CREB expression and downstream targets in ovarian granulosa cells in both CF and PCOS (Chen et al., 2012), we hypothesized that CFTR may be involved in the regulation of glucagon production by modulating p-CREB in α cells, and that defect or expression alteration of CFTR may dysregulate the glucagon levels, contributing to abnormal glucose levels as seen in CF and PCOS. We undertook the present study to test this possibility.

## Results

### Elevated glucagon levels in CFTR mutant mice

**Figure 1:**
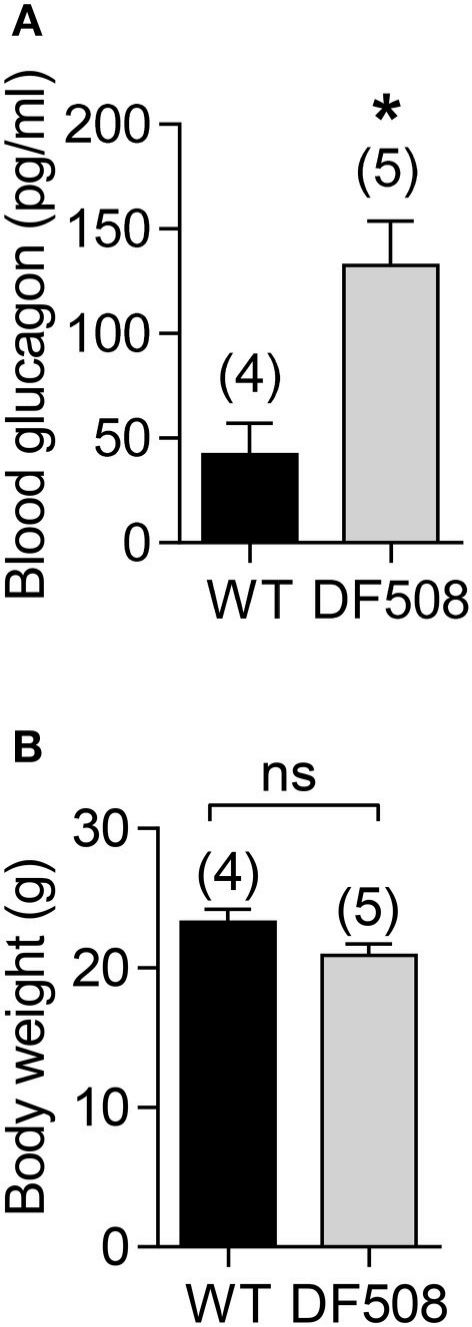
Elevated blood glucagon levels in DF508 mice. ELISA measurement of blood glucagon **(A)** and body weight **(B)** after fasting for 12 h in 12-week-old wildtype (WT) or DF508 mice. *t*-test, ^*^P < 0.05. ns, no significant difference. Number of animals is indicated above each bar.

To explore the role of CFTR in glucagon production, we performed *in vivo* study in a CFTR mutant mouse model with DF508, the most common mutation in CF patients (Cheng et al., 1995; Zeiher et al., 1995). DF508 mice showed a significant increased blood glucagon levels (Figure 1A) after 12 h fasting as compared to wildtype mice, although no significant difference in body weight (around 20 g) was found between DF508 and wildtype mice at the age of 12-week (Figure 1B). The increased glucagon levels observed in mice with CFTR mutation suggested a suppressive role of CFTR in glucagon production/secretion.

### Upregulation of glucagon and PC2 in CFTR mutant/inhibited mouse islets

**Figure 2:**
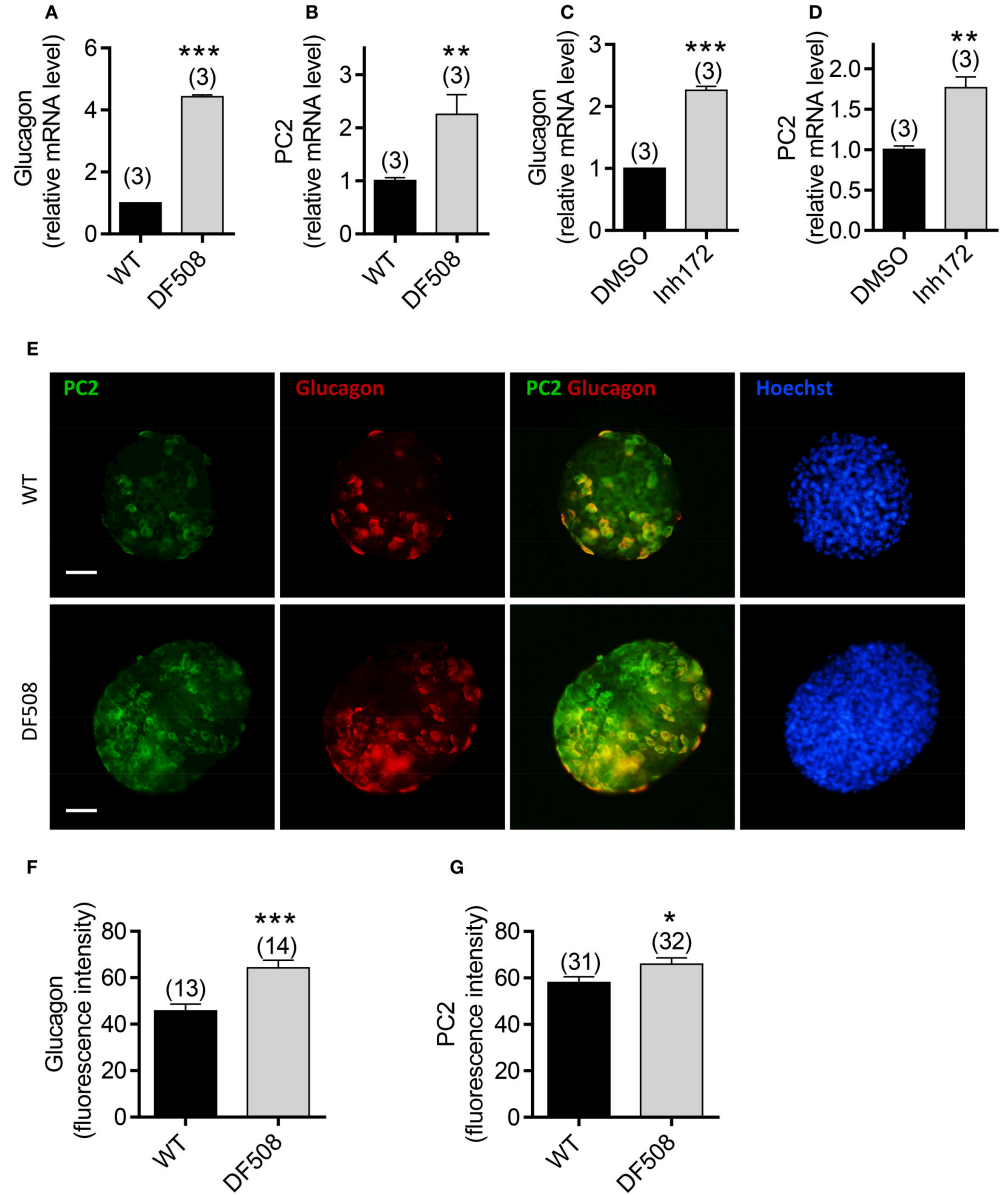
Effect of CFTR mutation or inhibition on glucagon expression in isolated mouse pancreatic islets. **(A–D)** Quantitative real-time PCR (qPCR) of glucagon **(A,C)** and PC2 **(B,D)** in wildtype (WT) and DF508 mouse islets **(A,B)**, or in WT mouse islets treated with CFTR inhibitor (Inh172,10 μM) or DMSO for 48 h **(C,D)**. *t*-test, ^**^P < 0.01, ^***^, *P* < 0.001. n is shown in each column. **(E–G)** Immunofluorescence labeling **(E)** with corresponding quantitation **(F,G)** for PC2 (green) and glucagon (red) in isolated islets from wildtype and DF508 mice. Scale bars, 100 μm. ^*^P < 0.05, ^***^P < 0.001, *t*-test. *n* is shown in each column.

To investigate the role of CFTR in glucagon synthesis specifically, we performed *ex vivo* study on isolated mouse islets. The results showed that the mRNA levels of glucagon and PC2 were significantly upregulated in isolated DF508 islets compared to wildtype islets (Figures 2A,B). Moreover, pretreatment of wildtype mouse islets with a CFTR inhibitor (Inh172, 10 μM) for 48 h resulted in a significant increase in mRNA expression of both glucagon and PC2 (Figures 2C,D) as compared to control islets treated with DMSO.

We also examined the protein expression of glucagon and PC2 in islets from DF508 mice by immunofluorescence. Consistent with the mRNA expression, the glucagon protein level was significantly higher in DF508 islets than wildtype, as indicated by quantification of fluorescence intensity (Figures 2E,F). Double labeling for PC2 and glucagon showed that PC2 was elevated in glucagon-positive α cells in DF508 islets compared to the wildtypes (Figures 2E,G). The observed upregulation of glucagon and PC2 at both mRNA and protein levels by CFTR mutation or inhibition in islet α cells further indicates a suppressive role of CFTR in glucagon production.

### Impaired glucagon levels in DHT-induced PCOS rat model

**Figure 3:**
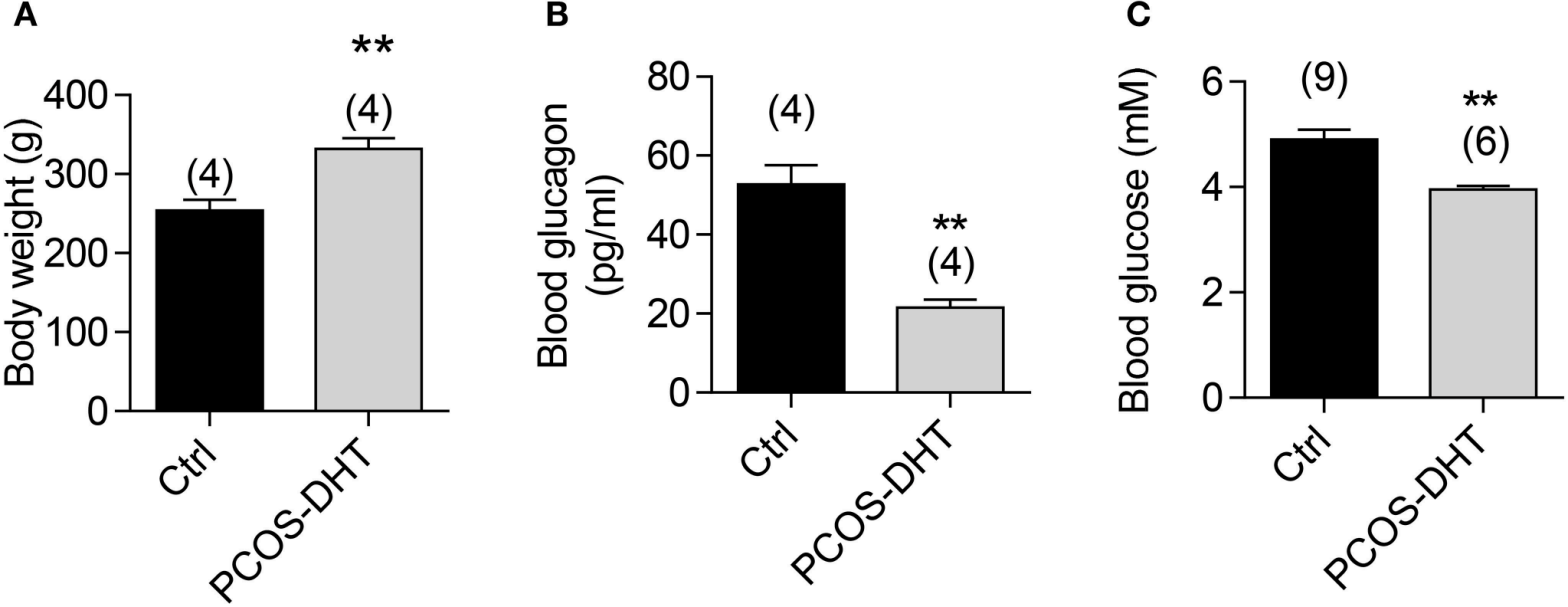
Glucagon and glucose levels in PCOS and control rats. Measurements of body weight **(A)**, blood glucagon **(B)** and blood glucose **(C)** after overnight fasting in PCOS and control rats after 7-week treatment of dihydrotestosterone (DHT) or vehicle (see section Materials and methods), respectively. ^**^P < 0.01, *t*-test. Number of animals is indicated above each bar.

We further tested the role of CFTR in regulating glucagon levels in PCOS, a condition known to have defective glucose metabolism and exhibit reciprocal relationship between glucagon and androgen levels (Golland et al., 1990). We established a rat PCOS model by chronic DHT treatment according to previous reports (see section Materials and Methods). Consistent with the obesity present in almost 50% PCOS women (Apridonidze et al., 2005), the DHT-treated rats showed greater body weight as compared to vehicle-treated control rats (Figure 3A). Moreover, significantly lower blood glucagon and glucose levels after fasting for 12 h were found in DHT-treated rats, as compared to the controls (Figures 3B,C). These findings revealed an impaired glucagon level in the PCOS model, accompanied with hypoglycemia in glucose-deprivation status.

### Higher CFTR and lower glucagon expression in PCOS islet α cells

**Figure 4:**
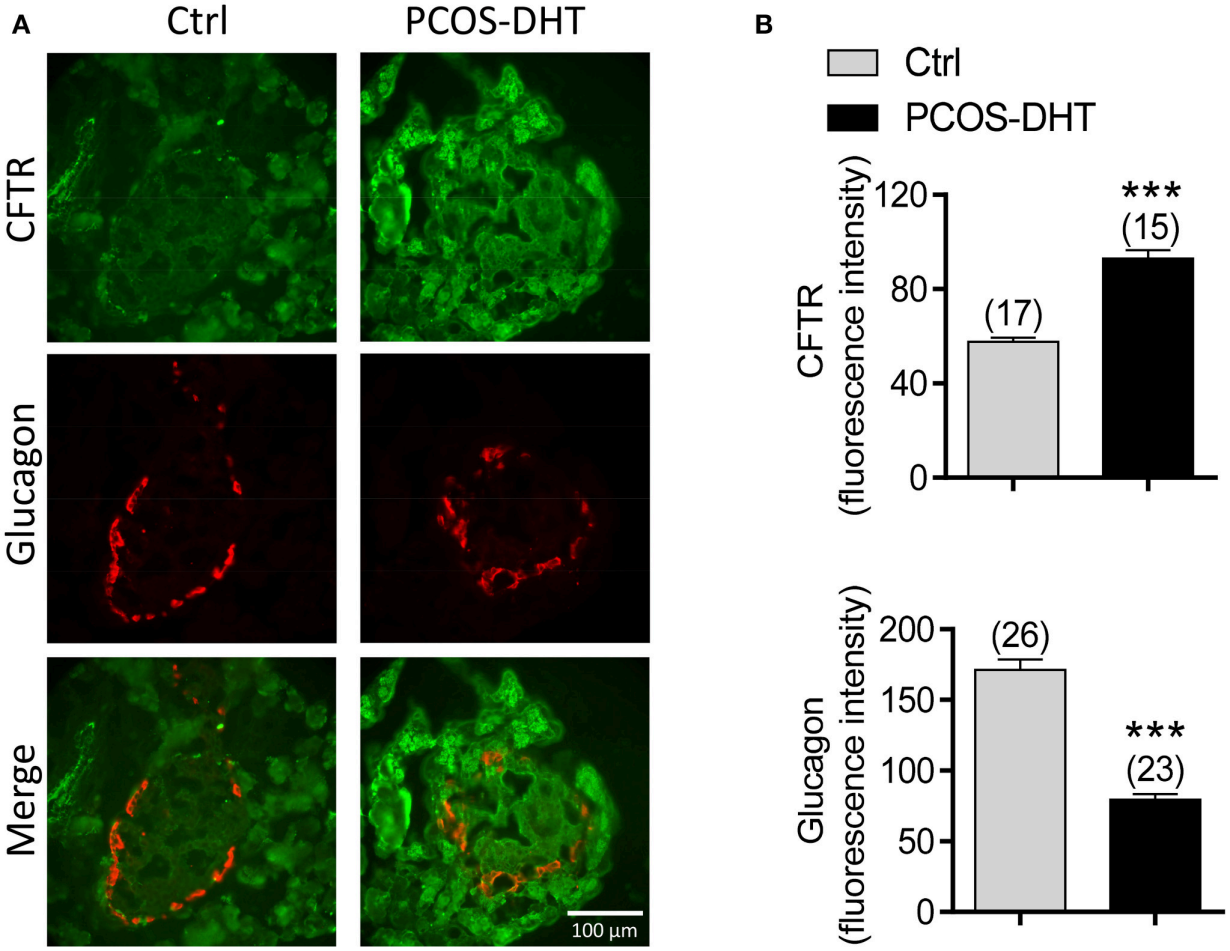
CFTR and glucagon expression in pancreatic α cells of PCOS and control rats. **(A,B)** Immunofluorescence labeling **(A)** with quantitation **(B)** for CFTR (green) and glucagon (red) in the pancreas of control (Ctrl) and PCOS rats after 7-week treatment with DHT (see section Materials and methods). ^***^P < 0.001, *t*-test. Scale bars, 100 μm. *n* is shown in each column.

Given that hyperandrogenism is a hallmark of PCOS (McCartney et al., 2006) and that CFTR can be upregulated by androgen (Xie et al., 2013), we suspected that the suppressed glucagon in PCOS could be due to androgen-induced upregulation of CFTR. We then sought to test whether CFTR expression is indeed upregulated in islet α cells in PCOS rats by immunofluorescence labeling for CFTR and glucagon. As shown in Figure 4A, in both control and PCOS pancreas, CFTR was found to be expressed in most of the cells in the pancreas, including α cells as indicated by positive glucagon labeling. The CFTR labeling was diminished by pre-incubation of CFTR antibody with a complementary peptide (Supplementary Figure 1), confirming that the labeling was specific for CFTR. Of note, quantification of fluorescence intensity showed that α cells (glucagon expressing cells) in PCOS pancreas exhibited significantly higher levels of CFTR, but significantly lower levels of glucagon, as compared to the controls (Figure 4B). Together, these results suggested that abnormally high expression of CFTR in islet α cells may be responsible for the impaired glucagon production in PCOS.

### DHT-induced CFTR upregulation and glucagon/PC2 downregulation in isolated mouse islets

**Figure 5:**
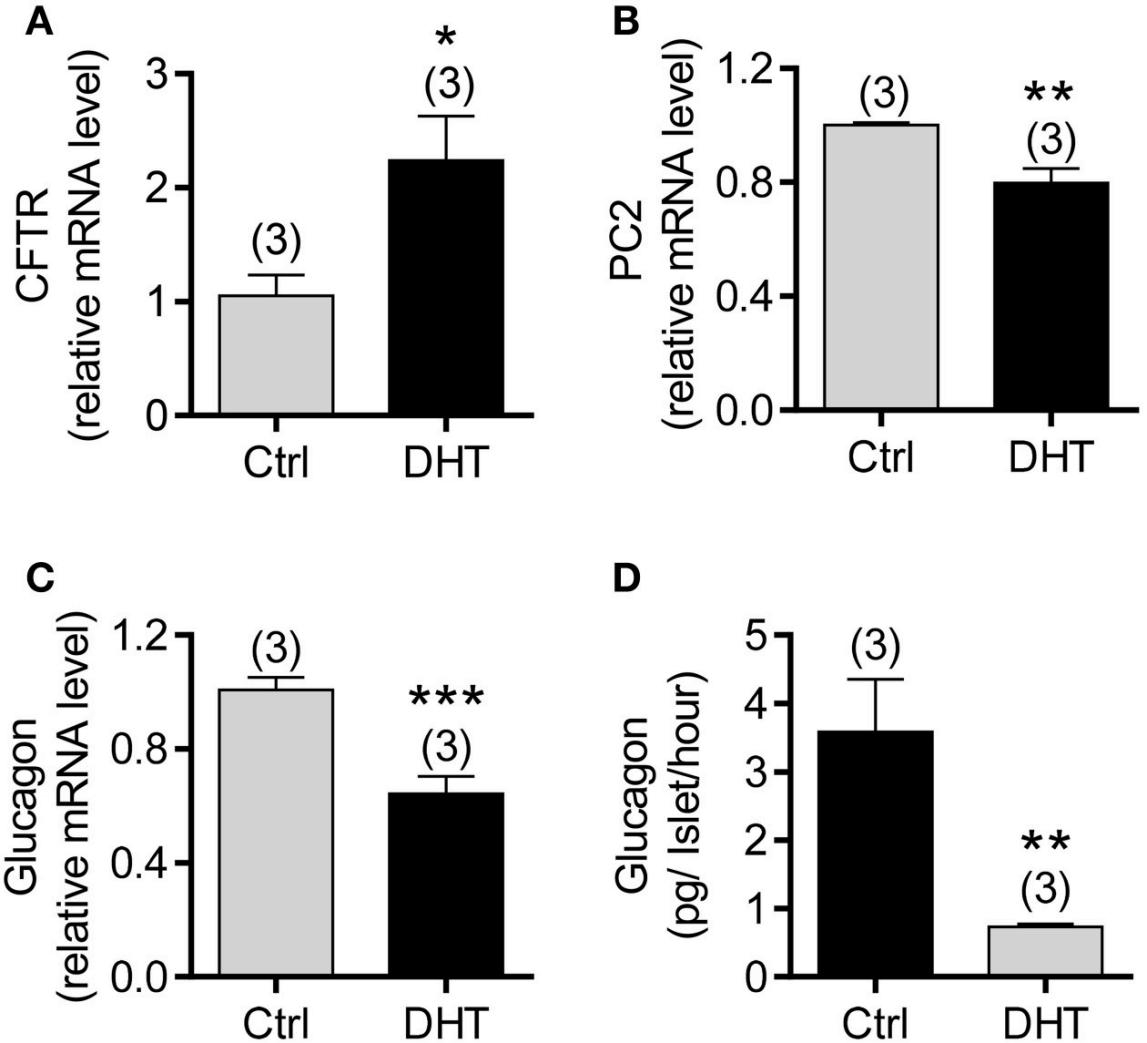
Effect of DHT on CFTR expression in α cells of isolated mouse islets. **(A–C)** qPCR of CFTR **(A)**, PC2 **(B)**, and glucagon **(C)** in mouse islets after exposed to DHT (100 nM) or ethanol as the vehicle control (Ctrl) for 48 h. ^*^P < 0.05, ^**^P < 0.01, ^***^P < 0.001, *t*-test. *n* is shown in each column. **(D)** ELISA of glucagon levels in the KRB solution with 1 mM glucose after 2 h incubation with isolated mouse islets. The islets were pretreated with DHT (100 nM) or the vehicle control (Ctrl) for 48 h. ^**^P < 0.01, *t*-test. The experiments were repeated at least three times.

We then asked whether increased CFTR expression and reduced glucagon in pancreatic α cells is due to high levels of androgen in PCOS. We treated isolated mouse islets with DHT (100 nM) for 48 h, and then examined mRNA expression of interested genes using qPCR. The results showed that the mRNA expression of CFTR was significantly upregulated, but glucagon and PC2 were downregulated in DHT-treated islets compared to the vehicle (ethanol)-treated islets (Figures 5A–C). Of note, in islets isolated from DF508 mice, the treatment with DHT (100 nM) did not change PC2 or glucagon mRNA levels (Supplementary Figure 2), suggesting the involvement of CFTR in the effect of DHT. At the protein level, we found that glucagon released from islets was significantly suppressed by DHT treatment in islets compared to that of the vehicle control group (Figure 5D). These results suggested that the DHT-induced upregulation of CFTR in α cells might be responsible for the reduced level of glucagon in PCOS.

### Involvement of CFTR in DHT-induced glucagon suppression in α cells

**Figure 6:**
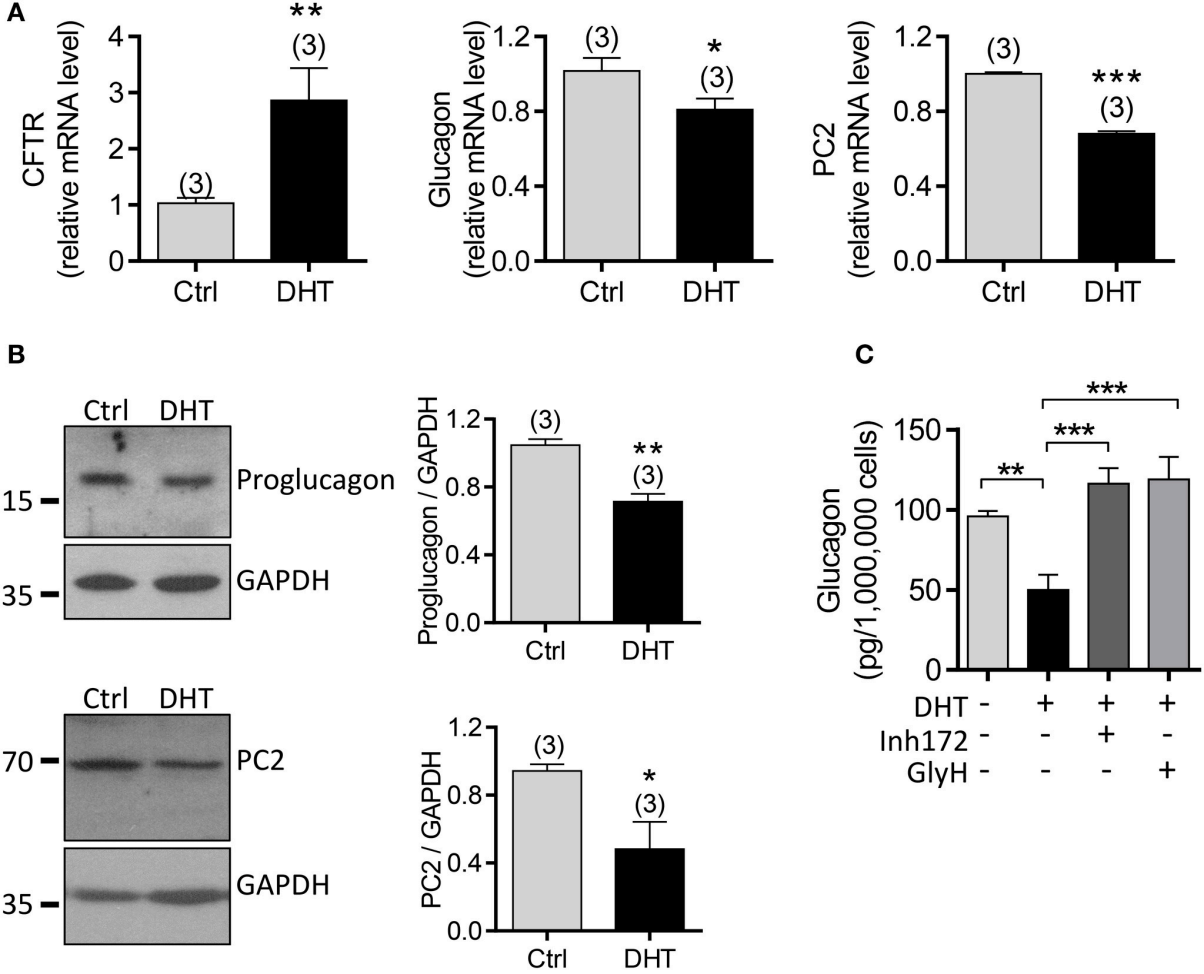
Involvement of CFTR in DHT-induced glucagon expression in αTC1-9 cells. **(A,B)** qPCR of CFTR, glucagon and PC2 **(A)** and western blot for proglucagon and PC2 **(B)**, with quantitation on the right) in αTC1-9 cells exposed to DHT (100 nM) or ethanol as the vehicle control (Ctrl) for 48 h. ^*^P < 0.05, ^**^P < 0.01, ^***^P < 0.001, *t*-test. n is shown in each column. **(C)** ELISA of glucagon levels in KRB solution containing 1 mM glucose after 1 h incubation with αTC1-9 cells, pre-incubated with DHT (100 nM), the CFTR inhibitors, Inh172 (10 μM) or GlyH-101 (GlyH, 10 μM) for 48 h. ^**^P < 0.01 ^***^P < 0.001, One way ANOVA. The experiments were repeated at least three times.

To exclude possible effect from other types of cells in islets, we next used the αTC1-9 cell line, a mouse α cell line, to explore the involvement of CFTR in mediating the DHT effect on glucagon production in α cells. qPCR results showed that mRNA level of CFTR was significantly increased, but glucagon and PC2 were reduced after exposure to DHT (100 nM) for 48 h in αTC1-9 cells (Figure 6A). Consistently, the protein levels of proglucagon, the precursor of glucagon, and PC2 were downregulated by DHT treatment (100 nM) in αTC1-9 cells (Figure 6B, full gel images shown in Supplementary Figure 4A). Importantly, pretreatment with CFTR inhibitors, Inh172 (10 μM) or GlyH-101 (10 μM) in αTC1-9 cells (Figure 6C) abolished the DHT-inducing glucagon-suppressing effect, indicating the involvement of CFTR in mediating the effect of DHT on suppressing glucagon production.

### CFTR-suppressed glucagon production through p-CREB in α cells

**Figure 7:**
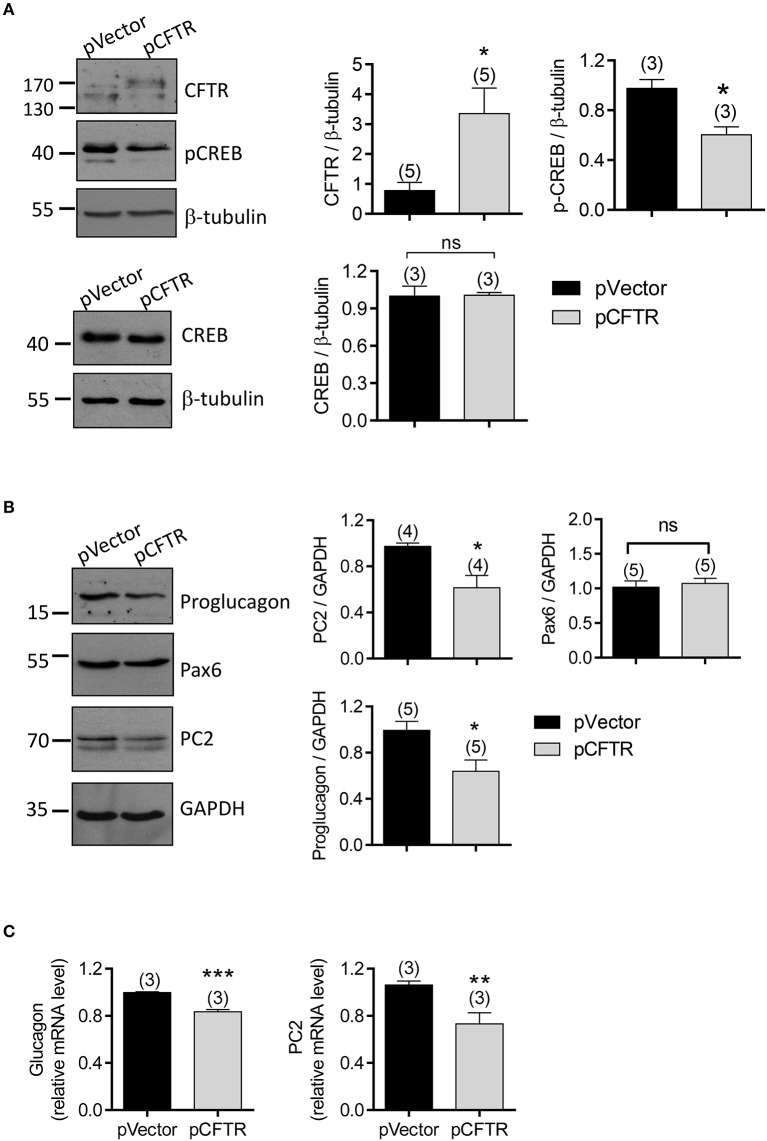
Involvement of p-CREB in CFTR-regulated glucagon production in αTC1-9 cells. **(A,B)** Western blot with quantitation (right) for CFTR, p-CREB, and CREB **(A)**, and proglucagon, Pax6 and PC2 **(B)** in αTC1-9 cells transfected with the empty vector plasmid (pVector) or pEGFP conjugated with full length CFTR (pCFTR). GAPDH or β-tubulin was used for loading control. ^*^P < 0.05, *t*-test. ns, no significant difference. n is shown in each column. **(C)** qPCR of glucagon and PC2 in αTC1-9 cells after transfected with pVector or pCFTR for 48 h. ^**^P < 0.01, ^***^P < 0.001, *t*-test, ns, not significant. The experiments were repeated for three times.

Since transcription factors, p-CREB and Pax6, are the essential components to bind with the enhancer region of glucagon-encoding gene (Gosmain et al., 2010, 2011, 2012), and the transcription of PC2 also depends on the presence of the cAMP-regulated enhancer (CRE) in its promoter and its binding with p-CREB (Espinosa et al., 2008), we suspected that CFTR may affect the glucagon production in α cells by regulating these important transcription factors. We transfected αTC1-9 cells with the vector containing full length CFTR (pCFTR), which resulted in overexpression of CFTR (Figure 7A), and significant reduction in protein levels of proglucagon and PC2, as compared to cells transfected with vector control (pVector) (Figures 7A,B, full gel images shown in Supplementary Figures 4B,C). In addition, significant lower p-CREB level was found in CFTR-overexpressing cells, whereas no significant effect on CREB or Pax6 was observed (Figures 7A,B). Consistently, qPCR results showed that CFTR overexpression reduced mRNA levels of both glucagon and PC2 in αTC1-9 cells (Figure 7C).

Intracellular Ca^2+^ has been known to play a key role in regulating hormone secretion, such as insulin by the β cells (Liang et al., 2014) and glucagon by the α cells (Chuang et al., 2011). Since phosphorylation of CREB can be activated by intracellular Ca^2+^ ([Ca^2+^]_i_) (Schwaninger et al., 1993), we further tested whether CFTR function affects intracellular calcium in α cells. Calcium imaging results showed that CFTR inhibitors, Inh172 (10 μM) or GlyH-101 (10 μM), induced a significantly higher [Ca^2+^]_i_ increase in CFTR overexpressing cells, as compared to control cells (Supplementary Figures 3A,B), indicating that CFTR inhibition results in calcium mobilization which may in turn activates p-CREB in α cells. Altogether, these findings suggested that CFTR normally suppresses glucagon synthesis through p-CREB at the transcription level, which may further affect the levels of genes involved in glucagon production or related enzyme conversion.

## Discussion

The present results have demonstrated that CFTR plays a suppressive role in regulating glucagon production in pancreatic α cells. This is supported by several lines of evidence. First, fasting blood glucagon levels, as well as mRNA expression levels of glucagon and PC2, are significantly higher in a CFTR mutant mice model, DF508 mice, compared to that of the wildtype. Second, similar results are also obtained from isolated islets showing elevated glucagon/PC2 mRNA and protein levels in islet α cells from DF508 mice or from wildtype islets with CFTR inhibition. Lastly, upregulation of CFTR either by DHT or by overexpression of CFTR result in glucagon/PC2 suppression in αTC1-9 cells; and the DHT-suppressed glucagon release can be reversed by inhibiting CFTR. Taken together, these results suggest that CFTR is a suppressor of glucagon production intrinsic to α cells since the use of an α cell line in the present study excludes the involvement of paracrine factors, such as GLP-1 (De Marinis et al., 2010), insulin (Llu et al., 1991), somatostatin (Strowski et al., 2000) or GABA (Xu et al., 2006). The fact that PC2 expression can also be affected by CFTR mutation/inhibition, as well as CFTR overexpression suggests that in addition to regulating glucagon expression directly, CFTR can also regulate glucagon production indirectly through PC2 to regulate the enzyme conversion of glucagon since effective glucagon is derived from the proglucagon by cleavage of PC2 (Rouillé et al., 1994; Piro et al., 2014). The presently demonstrated role of CFTR in regulation of glucagon-production related genes (i.e., glucagon and PC2) may underlie the pathogenesis of the glucagon level disruption seen in CF patients with diabetes (Hinds et al., 1991; Lanng et al., 1993; Edlund et al., 2017).

The present study has also demonstrated that CFTR regulates glucagon production at transcription level through p-CREB. p-CREB is considered a master transcription factor regulating glucagon (Gosmain et al., 2011) and PC2 (Espinosa et al., 2008). In fact, we observed that overexpression of CFTR results in decreased p-CREB, glucagon and PC2 in αTC1-9 cells (Figure 7), whereas upregulation of glucagon and PC2 by CFTR mutation or inhibition (Figure 2). Activation of CREB in the form of p-CREB is known to be regulated by cAMP, Ca^2+^ or both (Bito et al., 1996; Xu et al., 2011; Chen et al., 2012). In pancreatic islet cells, Ca^2+^ influx has been reported to promote the activation of CREB (Schwaninger et al., 1993). Of note, we have previously demonstrated that activation of epithelia Na^+^ channel (ENaC) promotes Ca^2+^ dependent phosphorylation of CREB, which in turn leads to the transcription of genes important for embryo implantation (Ruan et al., 2012; Sun et al., 2014). It thus suggests that ion channels may have the capacity of regulating Ca^2+^/CREB pathway. In this study, we have demonstrated that inhibiting CFTR triggers Ca^2+^ response in αTC1-9 cells (Supplementary Figures 3A,B), suggesting that normally CFTR may suppress p-CREB through its inhibitory action on Ca^2+^ response. Of note, another transcription factor Pax6 was not changed by CFTR overexpression in α cells (Figure 7), suggesting that Pax6 may not be involved in the CFTR-dependent regulation of glucagon. We previously showed that in germ cells (Xu et al., 2011) and granulosa cells (Chen et al., 2012), CFTR plays a role in promoting CREB phosphorylation/activation by transporting ${\text{HCO}}_{3}^{-}$ into the cells. Whereas, as presently observed in α cells, the effect of CFTR on CREB phosphorylation is rather suppressive, which could be explained by its inhibitory effect on intracellular Ca^2+^. As an ion channel, CFTR may contribute to membrane potential which may affect the open and close state of the voltage-sensitive Ca^2+^ channels, and thus the intracellular Ca^2+^ in α cells. In fact, a parallel study of ours (Huang et al., 2017) has shown that CFTR potentiates K_ATP_ channels contributing to a hyperpolarized membrane potential in α cells, which may underlie the effect of CFTR in suppressing intracellular Ca^2+^ and thus preventing CREB activation in α cells. Given the roles of CREB in transcription of a variety of genes (Lonze et al., 2002; Gosmain et al., 2011; Sun et al., 2014; Natalicchio et al., 2016), the presently demonstrated effect of CFTR on CREB may have implications in other organ systems, which await further investigation.

Another interesting finding of the present study is the concomitant upregulation of CFTR and downregulated glucagon/PC2 in DHT-induced PCOS animal/cell model. This observation further supports a suppressive role of CFTR in glucagon production. More interestingly, the present finding provides an explanation to the clinically observed inverse relationship between androgens and glucagon levels (Golland et al., 1990). The upregulation of CFTR observed in PCOS model and induced by DHT in α cells, together with the suppressed blood glucagon/glucose levels in PCOS model and DHT-suppressed glucagon release by α cells, which can be reversed by CFTR inhibitors, suggest that the impaired glucagon levels observed in PCOS patients are likely to be due to the hyperandrogenism-induced upregulation of CFTR, since hyperandrogenism is a hallmark of PCOS (Gambineri et al., 2002; Azziz et al., 2006). Of note, similar to the present finding, CFTR has been shown to be upregulated in the prostate with testosterone treatment (Xie et al., 2013). It is likely that the chronic exposure to high levels of androgen may result in upregulation of CFTR in α cells in PCOS patients, leading to impaired glucagon levels. Since glucagon is the major factor to recover blood glucose in glucose deprivation status, insufficient blood glucagon may potentially result in fatal hypoglycemia (Zoungas et al., 2010; Malmgren and Ahrén, 2015), which affects many PCOS patients (Altuntas et al., 2005; Barry et al., 2011). Hyperinsulinism, another hallmark of PCOS, often secondary to insulin resistance, is considered to contribute to abnormal glucose metabolism in PCOS, since insulin normally suppresses blood glucose level (Fica et al., 2008; Galluzzo et al., 2008). The present findings provide an alternative explanation for blood glucose metabolic disorder observed in PCOS via impaired glucagon production, as a result of hyperandrogenism-induced CFTR upregulation. Of interest, abnormal glucose levels are both observed in CF and PCOS patients (King and Rewers, 1993; Peppard et al., 2001; Moran et al., 2009). In light of the present findings, we propose that abnormal CFTR, either by its mutation as seen in CF or by its abnormal upregulation as seen in PCOS, may be one of the underlying causes for disordered glucose metabolism, leading to diabetes or insulin resistance seen in both diseases.

## Materials and methods

### Animals

*cftr*^*tm*1*Kth*^ mice (wildtype and DF508) (Zeiher et al., 1995) and Female Sprague Dawley rats were kept in a temperature-controlled room with a 12 h Dark-Light cycle, with food and water, *ad libitum* in the Laboratory Animal Service Center, the Chinese University of Hong Kong. The mice were supplied with regular diet with 20% DF508 mice surviving till adults. To establish a PCOS model, 21-day-old female rats were subcutaneously implanted on the back with silastic brand capsules (Dow Corning Corp., Midland, MI; continuous-release: 83 μg per day) filled with 7.5 mg of dihydrotestosterone (DHT, Dr. Ehrenstorfer, GmbH, Germany) or placebo capsules without bioactive molecule under sterile conditions. Blood was collected through tail vein from mice or rats. Animals were sacrificed by CO2 inhalation. All procedures were approved by the Animal Experimentation Ethics Committee of the Chinese University of Hong Kong. All methods were performed in accordance with the guidelines and regulations of the Chinese University of Hong Kong.

### Islet isolation

CFTR wildtype or DF508 mutant mice were killed at the age of 12–14 weeks and islets were isolated using the Type XI collagenase (Cat. C7657, Sigma, USA) as previously described (Li et al., 2009; Guo et al., 2014). Isolated islets were cultured in RPMI-1640 medium (Invitrogen, USA) with 10% fetal bovine serum (FBS), penicillin (100 IU/ml) and streptomycin (100 μg/ml).

### Cell culture

The mouse pancreatic α cell line, αTC1-9, was purchased from American Type Culture Collection (ATCC, USA) and cultured in Dulbecco's Modified Eagle's Medium (DMEM) containing 16.7 mM glucose with 10% FBS, penicillin (100 IU/ml) and streptomycin (100 μg/ml) at 37°C.

### CFTR overexpression

Cells were plated in 35 mm culture dishes at 70–80% confluence before transfection. Plasmids EGFP (pEGFP) or pcDNA3.1 conjugated with full-length human CFTR (pCFTR) were kindly provided by Professor Tzyh-Chang Hwang (University of Missouri-Columbia) (Yu et al., 2011; Xie et al., 2013). A total of 2.5 μg of plasmid was transfected using 6 μl lipofectamine 2000 (Cat. 11668-019, Invitrogen, USA). Forty-eight hours after transfection, cells were used to do following live experiments or protein/RNA collection.

### Glucagon secretion assay

Glucagon levels in mouse plasma or culture media were determined by ELISA (Cat. EIA-GLU-1, Raybiotech) following the manufacturer's instruction. After exposure to treatment reagents (DHT or CFTR inhibitor) or vehicle for 48 h in the full culture medium, cells or islets were pre-incubated in Krebs-Ringer buffer (KRB) containing (in mM): NaCl 119, KCl 4.7, CaCl_2_ 2.5, MgSO_4_ 1.2, KH_2_PO_4_ 1.2, NaHCO_3_ 25, HEPES 10 (pH 7.4) with 0.1% BSA for 15 min at 37°C, followed by 1 h (cells) or 2 h (Islets) test incubation in KRB with 1 mM glucose and 0.1% BSA at 37°C. Batches of 10–20 size-matched islets were selected under a dissecting microscope and used for each measurement by ELISA assay. To stimulation glucagon release, adrenaline (10 μM, David Bull Laboratories) and L-arginine (10 mM, Sigma) were added during the test incubation with islets.

### Quantitative real-time PCR (qPCR)

Total RNA of islets or cells was extracted by TRIzol reagent (Invitrogen, USA) according to the manufacturer's instruction. 0.5–1 μg total RNA was transcribed to complementary DNA (cDNA) using the cDNA ReverseTranscription kit (Cat. 4375575, Applied Biosystem, USA). One microliter cDNA was mixed with 0.25 μM forward primer, 0.25 μM reverse primer, 5 μl Bio SYBR Green Master mix (Takara, Japan) and dH_2_O up to 10 μl. qPCR was performed in triplicate on Applied Biosystem 7500 fast real-time PCR system (Thermo Fisher Scientific, USA). To calculate the relative transcriptional expression, the *Ct* values of genes were normalized by average Ct values of GAPDH as ΔCt. The relative transcriptional expression of interested genes was indicated with 2^(−−ΔΔ*Ct*)^. The sequences of forward and reverse primers were as follows: mouse Glucagon, 5′-TTC CCA GAA GAA GTC GCC ATT−3′, 5′- GGT GCT CAT CTC GTC AGA GAA−3′; mouse CFTR, 5′- GCT GAC ACT TTG CTT GCC CTG AG−3′, 5′- GCT TGC TGA TGG TCG ACA TAG GG−3′; mouse PC2, 5′- AGA GAG ACC CCA GGA TAA AGA TG−3′, 5′- CTT GCC CAG TGT TGA ACA GGT−3′, mouse GAPDH, 5′- AAC GAC CCC TTC ATT GAC−3′, 5′- TCC ACG ACA TAC TCA GCA C−3′.

### Western blot

Cells were lysed in RIPA buffer (150 mM NaCl, 50 mM Tris–Cl [pH 8.0], 1% NP-40, 0.5% sodium deoxycholate, 0.1% SDS, 1:100 protein inhibitors) for 30 min on ice. Supernatant was collected as total protein after centrifugation at 14,000 rpm for 30 min. Equal amounts of protein were separated by SDS-PAGE and transferred to nitrocellulose membranes (Bio-rad, USA) for immunoblotting. The protein bands were visualized by the enhanced chemo-luminescence assay (ECL, GE Healthcare, UK) following manufacturer's instructions and scanned by densitometer. Antibodies used are CFTR (Almone labs, ACL-006), proglucagon (Santa Cruz, sc-13091), PC2 (Santa Cruz, sc-30166), pax6 (Santa Cruz, sc-53106), p-CREB (CST, 9198), CREB, (CST, 9197), β-tubulin (Santa Cruz, sc-9104), and GAPDH (Santa Cruz, sc-47724).

### Immunofluorescent staining

Pancreas of rats were fixed in 4% PFA, embedded with O.C.T. Compound (Sakura Finetek USA Inc.) and cut into cryosections. Frozen sections were incubated in a boiled water bathed-alkaline buffer (10 mM Tris base, 1 mM EDTA, 0.05% Tween 20, pH 9.0) for 7 min, cooled down to room temperature, and treated with 1% SDS for 4 min. For whole islet staining, islets were transferred from one solution to another solution by picked up with pipette tips under dissecting microscope. The sections or islets were blocked by 1% BSA in PBS for 60 min at room temperature, followed by primary antibody incubation overnight at 4°C. Primary antibodies were: CFTR (Almone labs, ACL-006), glucagon (Abcam, ab10988), PC2 (Santa Cruz, sc-30166). In experiments indicated, the CFTR antibody (Almone labs, ACL-006) was incubated with a complimentary peptide (1: 2) (Almone labs) for 1 h before used for staining. After washed for three times with PBS, fluorophore-conjugated secondary antibodies were applied for 1 h, then Hoechst 33342 was used for nucleus staining. Fluorescent images were captured with fluorescence microscopy (Nikon) or confocal microscopy (ZEISS). Quantitation of relative fluorescence intensity of interested proteins was analyzed with Metamorph.

### Intracellular calcium measurement

The αTC1-9 cells were loaded with Fura-2-AM (3 μM) in a bath solution (Margo-Ringer buffer) with 10 mM glucose for intracellular calcium monitoring as previous described (Guo et al., 2014). Intracellular calcium change was reflected by the change in the ratio of 340/380 fluorescent signal intensity of Fura-2.

### Statistical analysis

Data are means ± S.E.M. Student's unpaired *t*-test was used for comparison between two groups, and One-way ANOVA was used for three or more groups. Differences between groups was analyzed using Prism 5.0. A *p*-value < 0.05 was considered statistically significant.

## Author contributions

HCC and JHG conceptualized. HCC, YCR, JHG, and WQH designed. WQH, JHG, YCR, CY, and MKY performed the experiments and data analysis. JYL, CY, YGC, and FYD established the PCOS model and provided consultancy. WQH wrote the manuscript, HCC and YCR revised.

### Conflict of interest statement

The authors declare that the research was conducted in the absence of any commercial or financial relationships that could be construed as a potential conflict of interest.

## Supplementary material

Supplementary Figure 1 CFTR and glucagon expression in pancreatic α cells of PCOS and control rats. Confocal images of immunofluorescence staining for CFTR (green) and glucagon (red) in the pancreas of control (Ctrl, **A,C**) and PCOS **(B,D)** rats after 7-week treatment with DHT. The CFTR antibody was pre-incubated with a complimentary peptide for 30 min before used for staining in C&D. Scale bar, 50 μm.

Supplementary Figure 2 Effect of DHT on DF508 mouse islets. qPCR of glucagon **(A)** and PC2 **(B)** in wildtype (WT) and DF508 mouse islets after exposed to DHT (100 nM) or ethanol as the vehicle control for 48 h. ^*^*P* < 0.05, ^**^*P* < 0.01, *t*-test. The experiments were repeated three times.

Supplementary Figure 3 Effect of CFTR inhibition on intracellular Ca^2+^ in αTC1-9 cells. Representative time-course recording **(A)** and corresponding statistics **(B)** of intracellular Ca^2+^ measurement using Fura-2 in αTC1-9 cells 48 h after transfected with pVector or pCFTR in response to the CFTR inhibitor, Inh172 (10 μM) or GlyH (10 μM) in 10 mM glucose bath solution. Number of measurement was indicated in each column. ^***^*P* < 0.001, one-way ANOVA.

Supplementary Figure 4 Full gel images of Figure 6B **(A)**, Figure 7A **(B)**, and Figure 7B **(C)**.
