# Supplementary figures and images for: Abnormal CFTR Affects Glucagon Production by Islet α Cells in Cystic Fibrosis and Polycystic Ovarian Syndrome
Source: Front Physiol. 2017 Nov 17;8:835. doi: 10.3389/fphys.2017.00835 (PMC5698272; doi:10.3389/fphys.2017.00835)

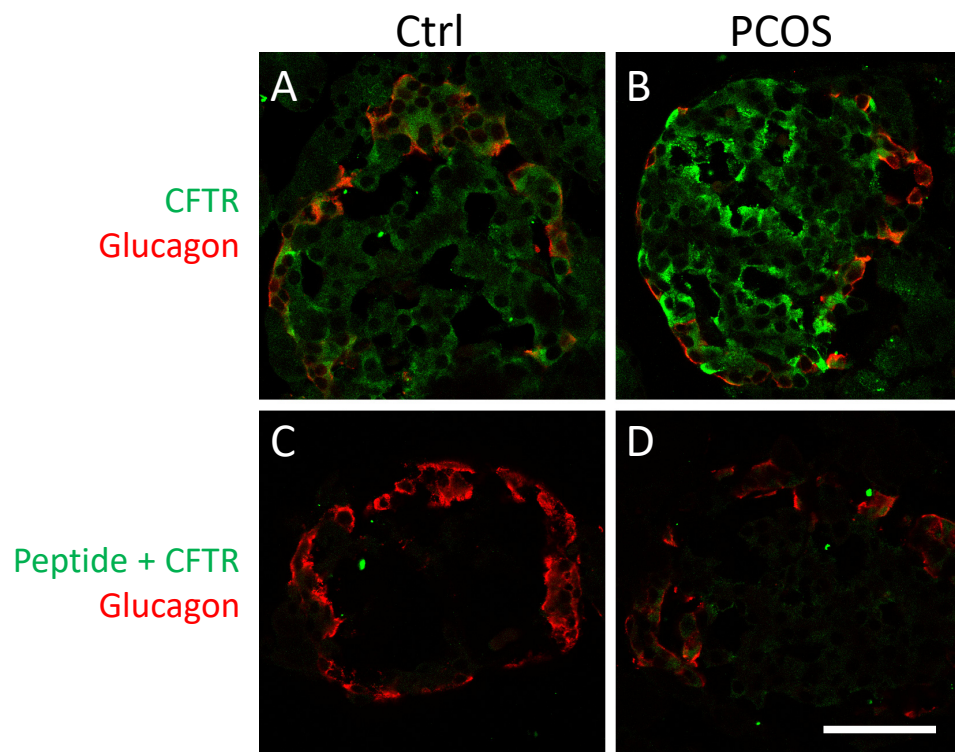

Suppl. Figure 1

A

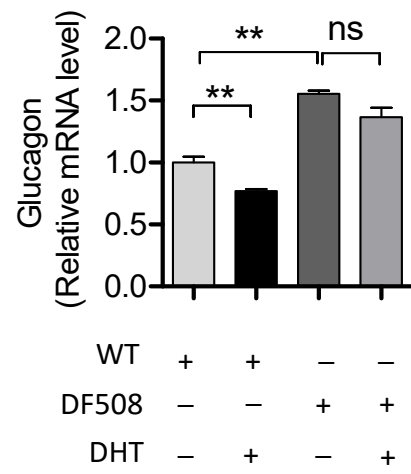

B

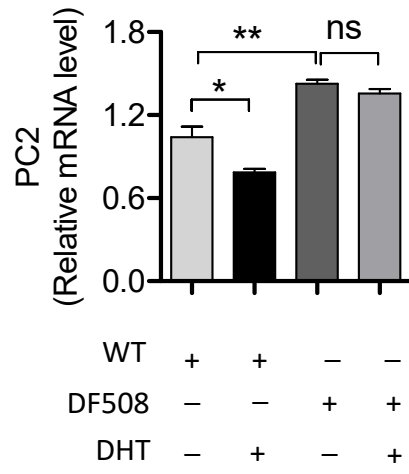

A

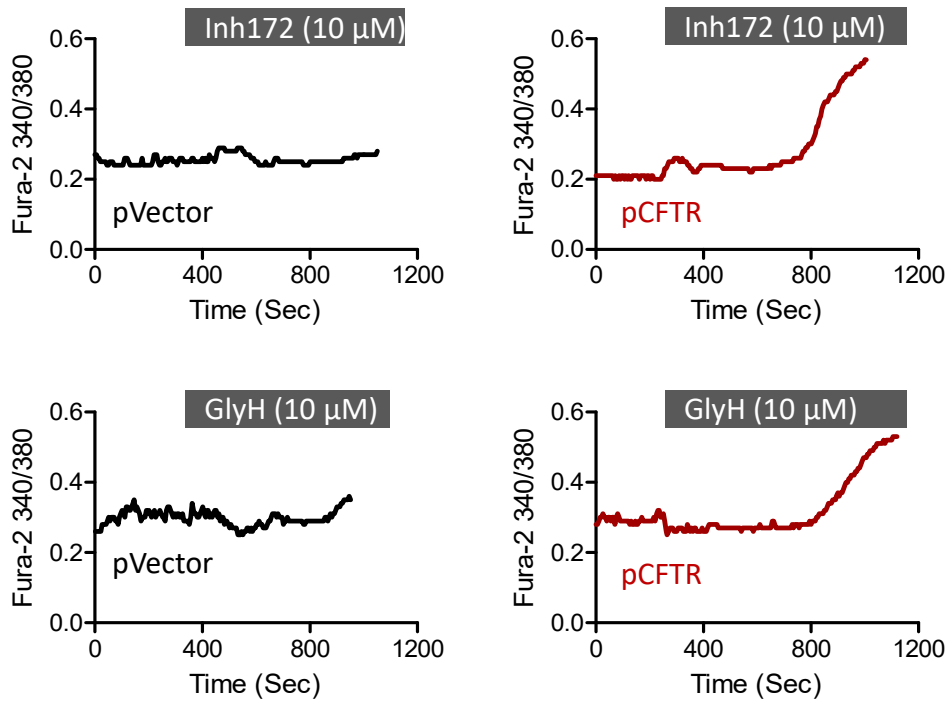

B

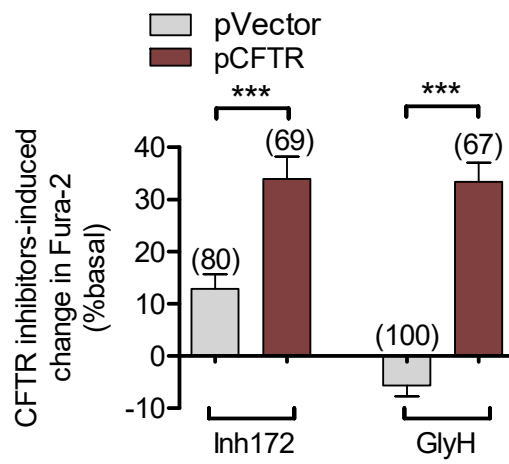

A

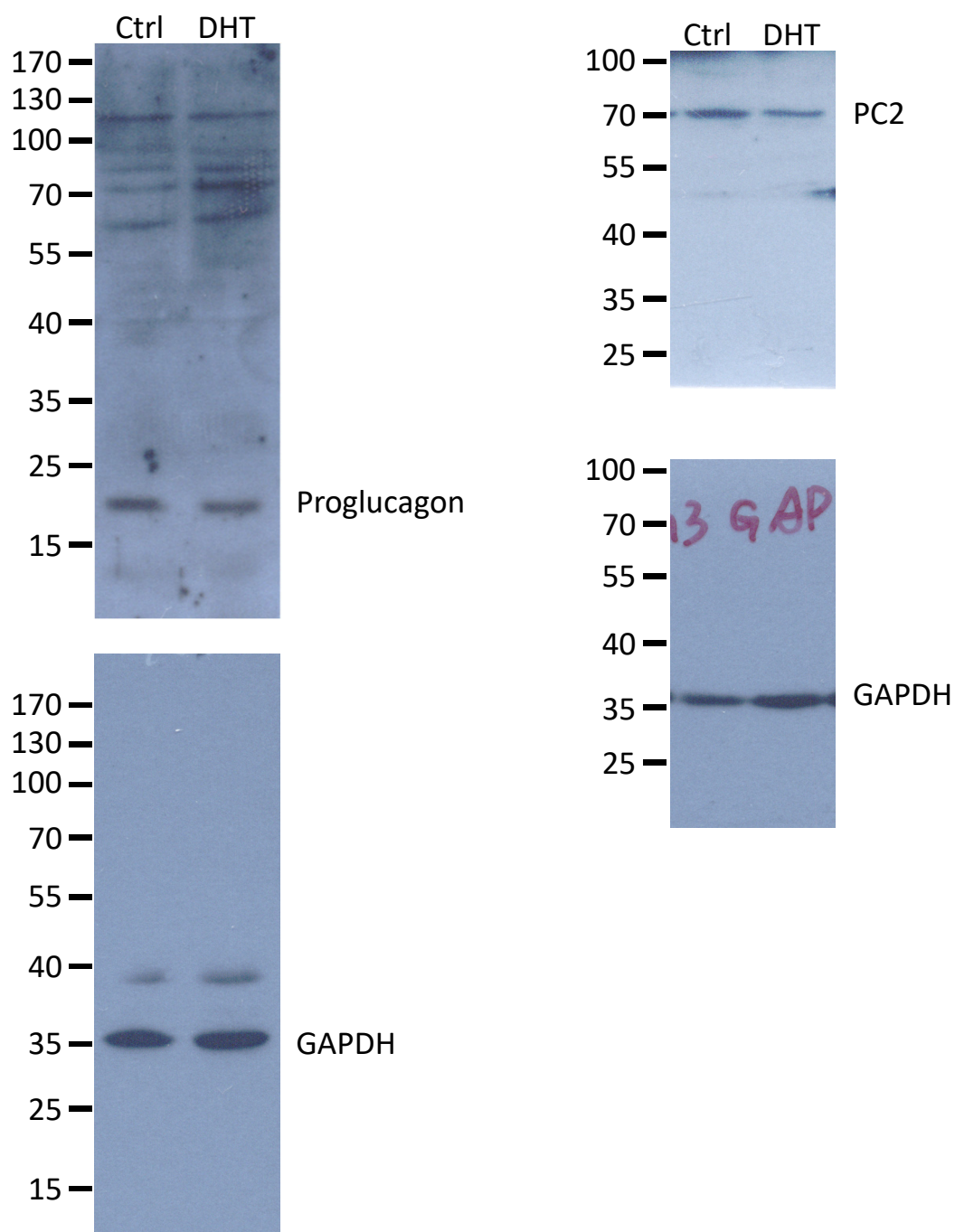

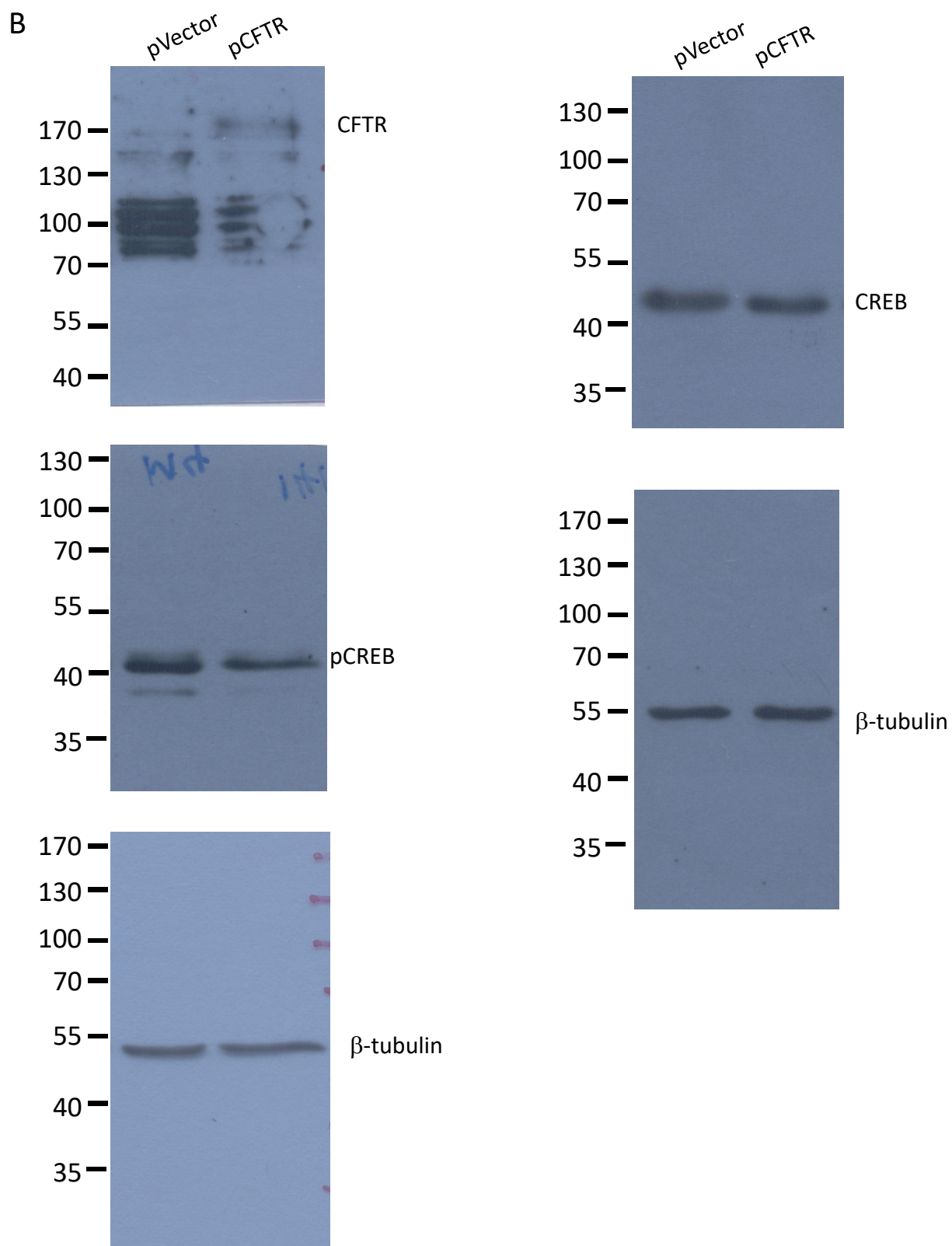

Suppl. Figure 4

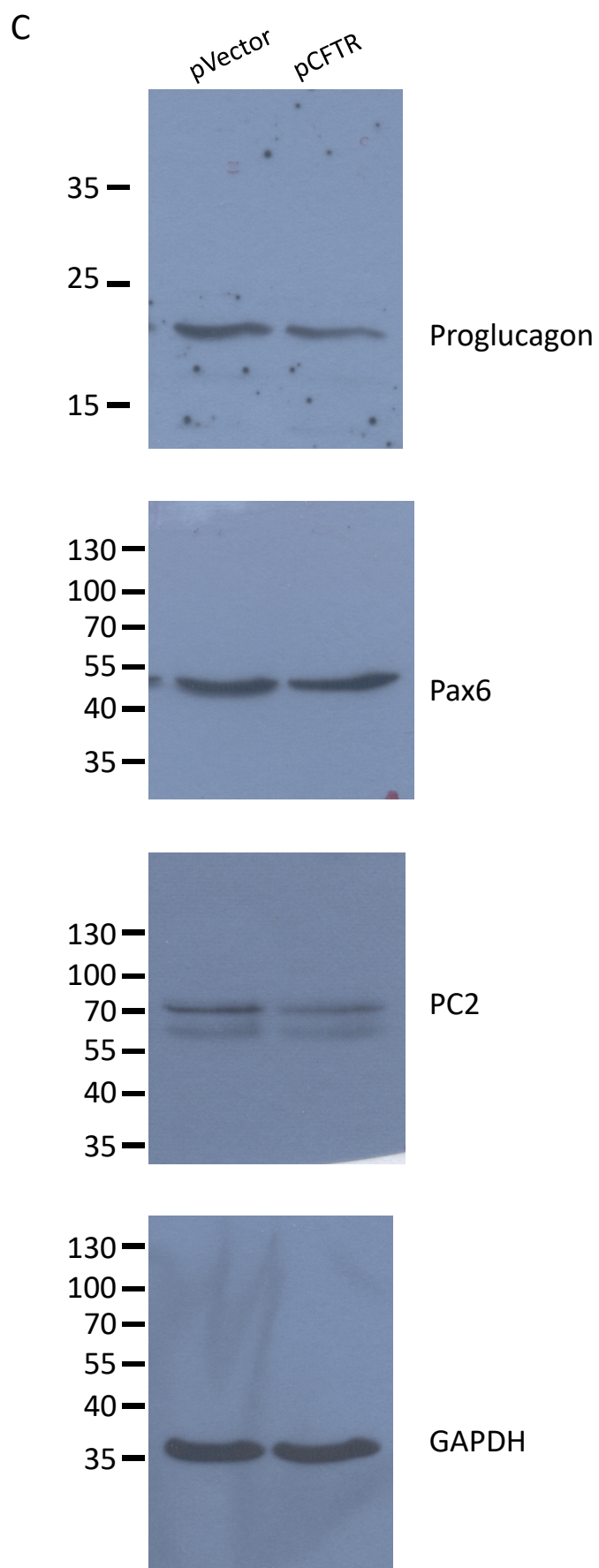

Supplement: Supplementary Figure 1 — CFTR and glucagon expression in pancreatic α cells of PCOS and control rats. Confocal images of immunofluorescence staining for CFTR (green) and glucagon (red) in the pancreas of control (Ctrl, A,C) and PCOS (B,D) rats after 7-week treatment with DHT. The CFTR antibody was pre-incubated with a complimentary peptide for 30 min before used for staining in C&D. Scale bar, 50 μm. [file DataSheet1.PDF]
